# The Proteomic Profile of Hereditary Inclusion Body Myopathy

**DOI:** 10.1371/journal.pone.0016334

**Published:** 2011-01-31

**Authors:** Ilan Sela, Irit Milman Krentsis, Zipora Shlomai, Menachem Sadeh, Ron Dabby, Zohar Argov, Hannah Ben-Bassat, Stella Mitrani-Rosenbaum

**Affiliations:** 1 Goldyne Savad Institute for Gene Therapy, Hadassah Hebrew University Medical Center, Jerusalem, Israel; 2 Laboratory of Experimental Surgery, Hadassah Hebrew University Medical Center, Jerusalem, Israel; 3 Department of Neurology, Wolfson Medical Center, Holon, Israel; 4 Department of Neurology, Hadassah Hebrew University Medical Center, Jerusalem, Israel; McMaster University, Canada

## Abstract

Hereditary inclusion body myopathy (HIBM) is an adult onset, slowly progressive distal and proximal myopathy. Although the causing gene, GNE, encodes for a key enzyme in the biosynthesis of sialic acid, its primary function in HIBM remains unknown. The goal of this study was to unravel new clues on the biological pathways leading to HIBM by proteomic comparison. Muscle cultures and biopsies were analyzed by two dimensional gel electrophoresis (2-DE) and the same biopsy extracts by isobaric tag for relative and absolute quantitation (iTRAQ). Proteins that were differentially expressed in all HIBM specimens versus all controls in each analysis were identified by mass spectrometry. The muscle cultures 2-DE analysis yielded 41 such proteins, while the biopsies 2-DE analysis showed 26 differentially expressed proteins. Out of the 400 proteins identified in biopsies by iTRAQ, 41 showed altered expression. In spite of the different nature of specimens (muscle primary cultures versus muscle biopsies) and of the different methods applied (2D gels versus iTRAQ) the differentially expressed proteins identified in each of the three analyses where related mainly to the same pathways, ubiquitination, stress response and mitochondrial processes, but the most robust cluster (30%) was assigned to cytoskeleton and sarcomere organization. Taken together, these findings indicate a possible novel function of GNE in the muscle filamentous apparatus that could be involved in the pathogenesis of HIBM.

## Introduction

Hereditary inclusion body myopathy (HIBM or IBM2; OMIM 600737) constitutes a rare autosomal recessive neuromuscular disorder characterized by slowly progressive distal and proximal muscle weakness of the limbs [1]. HIBM is most common among the Jewish-Persian community with a prevalence of 1∶1500. However, in Japan there is an important cluster of patients with the same disorder, known there as distal myopathy with rimmed vacuoles (DMRV) and affected single non Jewish families from different origins can be found worldwide. The initial symptom, altered gait, appears usually during the third decade of life due to foot dorsiflexion muscles involvement. Later in the course of the disease other muscles are involved, however for unknown reason the quadriceps is relatively spared. A typical HIBM muscle pathology includes cytoplasmic rimmed vacuoles and cytoplasmic or nuclear 14 to 18nm tubulofilamentous inclusions that give the disease its name [2]. The molecular basis of the disease was found to be mutations in *GNE*, the gene encoding the enzyme UDP-N-acetylglucosamine 2-epimerase/N acetylmannosamine kinase (UDP-Glc-NAc 2-epimerase/ManNAc kinase) [3]. In Jews of Persian descent, a single homozygous missense mutation was identified, however in patients from non Jewish families of various origins, different mutations, mostly missense compound heterozygotes, were found in all exons of GNE, in both domains of the protein [4], [5]. GNE is the rate-limiting enzyme in the biosynthetic pathway of sialic acid and catalyzes the first two steps by each one of the two distinct functional domains [6]. Sialic acids are the most abundant terminal monosaccharides on glycoproteins and glycolipids in eukaryotic cells. They comprise a family of more than 50 naturally occurring carboxylated amino sugars with a scaffold of nine carbon atoms [7]. Sialic acids influence adhesion processes which play an important role in many cellular functions, such as cell migration, transformation of tissues, inflammation, wound healing and metastasis [8]–[10]. In spite of extensive experimental work in the last decade, the contribution of *GNE* mutations to the HIBM phenotype remains unresolved and the issue of hyposialylation in HIBM muscles is still controversial. The goal of this study was to compare the proteomes of HIBM primary muscle cultures and biopsies to controls by 2 dimensional polyacrylamide gel electrophoresis (2-D PAGE) and isobaric tag for relative and absolute quantitation (iTRAQ) analyses in an attempt to identify potential proteins that may be involved in the HIBM phenotype and therefore unravel biological pathways affected by mutations in GNE.

## Materials and Methods

### Ethics Statement

Muscle samples were collected with the informed consent of the participants. These studies were approved by the Institutional Review Board of Edith Wolfson Medical Center, Holon, Israel.

### Muscle biopsies

All muscle biopsies were collected and immediately frozen in liquid nitrogen until used for protein extraction. An additional muscle specimen was placed in PBS and used for the establishment of primary myoblast culture for some individuals (Table 1). The normal muscle specimens were collected from consenting individuals who had undergone either muscle biopsy which eventually was diagnosed as normal or orthopedic surgery. HIBM patients were clinically and genetically diagnosed, most of them of Jewish Persian descent carrying the homozygous M712T mutation in GNE, unless otherwise noted. Since high variability can be found in many parameters among muscle and muscle cultures from different individuals, we matched appropriate control sample to each HIBM sample, by age (all young adults), gender and muscle type, as closely as possible (Tables 1, 2). Biopsies of patients were taken from clinically affected muscles.

**Table 1:** Characteristics of muscle cultures used in 2-DE analysis.

Status	Muscle specimen	Age (years)	Gender	Culturea)
HIBM	Deltoid	26	Male	MS-17^1^
HIBM	Deltoid	29	Male	MS-20^2^
HIBM	Gastrocnemius	30	Male	MS-312^3^
Control	Deltoid	27	Male	N-17^1^
Control	Deltoid	33	Male	N-20^2^
Control	Deltoid	46	Male	MS-35^3^

a) Matched pairs are labeled with the same number: 1, 2 or 3.

**Table 2:** Characteristics of Muscle biopsies used for 2-DE and iTRAQ analyses.

Status	Muscle specimen	Age (years)	Gender	Biopsya)
HIBM	Deltoid	48	Male	B-147^1^
HIBM	Tibialis anterior	22	Female	B-220^2^
HIBM	Deltoid	35	Female	B-171^3^
Control	Deltoid	48	Male	B-270^1^
Control	Deltoid	22	Male	B-181^2^
Control	Deltoid	32	Male	B-190^3^

a) Matched pairs are labeled with the same number: 1, 2 or 3.

### Analysis of muscle cultures by 2-D PAGE

All chemicals were obtained from SIGMA (St. Louis, MO, USA). DME medium, Ham's F-10 medium, FBS, horse serum, PBS, Trypsin, Penicillin, Streptomycin and Glutamine were obtained from Biological Industries (Beit Haemek, Israel). Fresh biopsies were processed as previously described [11]. To initiate differentiation, growth medium was replaced by DME medium containing 2% HS, referred as differentiation medium. Myoblasts were differentiated for at least 5 days. Sialic acid uptake was avoided by growing the cells in serum-free medium (DCCM, Biological Industries) for 2–3 days prior to harvesting. Differential cells were harvested at 70% confluency by 2 mM EDTA in order to avoid digestion of membrane proteins by trypsin. Cell pellets were resuspended in 0.5 mL lysis buffer (10 mM Tris-HCl, pH 7.4, 1 mM EDTA and 1.4 mM PMSF) and disrupted by sonication. Cell debris and protein aggregates were removed by centrifugation at 20000×g for 30 min at 4°C. The protein concentration was determined using the Bradford method (Sigma) with the Protein Assays kit (Bio-Rad, CA, USA). The supernatants containing the proteins were lyophilized and sent to the Maiman Institute for Proteome Research at Tel Aviv University, Israel. Isoelectric focusing was performed with IPG-strips, 18 cm, pH 4–7 (Amersham Biosciences, NJ, USA). Samples were loaded by rehydration for 24 h in a solution containing 8 M urea, 2 M thiourea, 1% w/v CHAPS, 20 mM DTT and 0.5% v/v Pharmalyte 4–7. The isoelectric focusing was performed with the MultiphorII unit (Amersham Biosciences) employing the following voltage profiles [12]: linear increase from 0 to 500 V for 2500 Vh, 500 V for 2500 Vh, linear increase from 500 to 3500 V for 10000 Vh and a final phase of 3500 V for 35000 Vh. After consecutive equilibration of the gels in solutions containing DTT and iodoacetamide as suggested by Görg *et al.* [13] the separation in the second dimension was performed in polyacrylamide 20×20 cm gels of 12.5% on a 2-DE system (Genomic Solutions, Chelmsford, MA, USA). Analytical and preparative gels were loaded with 350 µg of crude protein extract and the gels were stained with colloidal Coomassie.

### Peptide mass sequencing and protein identification

Protein identification was accomplished according to established protocols [14]. Briefly, protein spots were excised from Coomassie Blue stained gels, the gel pieces were washed in a 200 mM NH_4_HCO_3_-50% acetonitril solution for 30 minutes at 37°C. The solution was discarded and the gels were dried in a speed vac for 30 minutes. The gels were rehydrated in a solution of 20 µg/mL trypsin (Promega, Madison, WI, USA) and the proteins were digested for 16 h at 37°C. Peptides were extracted from the gel by diffusion in 10% acetonitril and desalted on micro-columns containing a 1∶1 mixture of POROS R2–50 and Poros Oligo R3 (Perseptive BioSystems). The peptides were eluted with 70% (v/v) acetonitril-double distilled water saturated with α-cyano-3-hydroxycinnamic acid onto a sample plate for MALDI-MS. Peptide masses were determined in the positive ion reflector mode in a Voyager-DE STR mass spectrometer (Applied Biosystems, CA, USA) with internal calibration. Peptide mass fingerprints were compared to databases using the MS-Fit program (http://prospector.ucsf.edu). The searches took into consideration oxidation of methionine, pyroglutamic acid formation at the N-terminal glutamine and modification of cysteine by carbamidomethylation or acrylamide, as well as partial cleavage leaving one internal cleavage site [14].

### Western analysis

For Western blot analysis, lysates equivalent to 25 µg of total protein were fractionated by denaturing sodium dodecyl sulfate 7.5% polyacrylamide gel electrophoresis and transferred to nitrocellulose (Schleicher and Schuell, Dassel, Germany) by standard electroblotting techniques. After 1 h blocking in 3% BSA/PBS-Tween (0.01%), membrane was washed 3 times with PBS-Tween (0.01%) and incubated for 1 h with primary antibody diluted in blocking buffer. To validate 2-D PAGE results the following primary Abs were used: mouse monoclonal anti troponin T1 (1∶1000; Chemicon, CA, USA), mouse monoclonal anti VCP (1∶300; BD Biosciences, CA, USA), mouse monoclonal anti vimentin (1∶1000; Sigma), mouse monoclonal anti N-CAM (1µg/ml; Invitrogen, CA, USA), goat polyclonal anti HSP 27 (M-20) and rabbit polyclonal anti lamin a/c (1∶500; Santa Cruz Biotechnology, CA, USA). Detection was performed by enhanced chemiluminescence after incubation with HRP-conjugated antibodies (1∶80000; Jackson ImmunoResearch, West Grove, PA, USA).

### Analysis of biopsy specimens by 2-D PAGE

Total protein was extracted from biopsies by TRIzol™ (Sigma) according to manufacturer protocol. 50µg of each protein extract were transferred to a new tube and used later for iTRAQ analysis and the remained extract was prepared for the 2-D PAGE analysis. Both analyses were done at the Smoler Proteomics Center at the Technion, Haifa. The remaining protein sample was resuspended in a sample solubilization buffer containing 7 M urea, 2 M thiourea, 2% w/v CHAPS, 65 mM DTT, 0.3% v/v ampholyte 3–10, 0.1% v/v ampholyte 6–11, 0.1% v/v ampholyte 4–7 and 5 µL Bromo-Phenol-Blue (BPB). Protein amount was quantified using a modified Bradford analysis. The IPG strip (3–11 NL, Immobiline DryStrip 18 cm, Amersham Biosciences) was rehydrated with 400 µg protein in 350 µL of the protein sample. The rehydration was performed for 1 h with no voltage followed by 12–16 h at 50 mV. Isoelectric focusing was then performed for 1 h at 200 V, 1 h at 500 V, 1 h at 1000 V, 30 min linear increase to 8000 V and up to 12 h at 8000 V to the final value of 95000 VH. Following isoelectric focusing each strip was equilibrated in 100 mg DTT in 10 mL of equilibration buffer containing 6 M urea, 30% w/v glycerol and 2% SDS in 0.05 M Tris-HCl buffer (1.5 M Tris-HCl and 0.4% w/v SDS), pH 8.8 with 10 µL BPB for 20 min. A second equilibration step was performed for 20 min with 400 mg IAA in 10 mL of the above equilibration buffer. After equilibration, each sample of the immobilized pH gradient strips was loaded onto Tris-SDS gel 7–15%, 20×20.5 cm along with “Dual Color” molecular weight marker (Bio-Rad, CA, USA). The proteins entered the gel in 100 V for ∼5 h and ran over night at 30 V. Each Gel sample was stained with Imperial protein staining solution (Pierce, IL, USA). Images of the gels were acquired using Microtek's ScanMaker 9800XL and analyzed using PDQUEST 7.3.1 software (Bio-Rad).

### In gel proteolysis and mass spectrometry analysis

Selected differential spots were cut from the gel. The proteins in the gel were trypsinized (Promega) at a 1∶100 enzyme to substrate ratio. The resulting tryptic peptides were resolved by reverse-phase chromatography on 0.075×200 mm fused silica capillaries (J&W; Agilent technologies, Santa Clara, CA, USA) packed with Reprosil reversed phase material (Dr Maisch GmbH, Germany). The peptides were eluted with linear 65 minutes gradients of 5 to 45% and 15 minutes at 95% acetonitrile with 0.1% formic acid in water at flow rates of 0.25 µL/min. Mass spectrometry was performed by an ion-trap mass spectrometer (Orbitrap; Thermo Fisher Scientific, Waltham, MA, USA) in a positive mode using repetitively full MS scan followed by collision induces dissociation (CID) of the 7 most dominant ion selected from the first MS scan. The mass spectrometry data was clustered and analyzed by using the Sequest software [15] and Pep-Miner [16] searching engine against the Human part of the NR-NCBI database.

### Analysis of biopsy specimens by iTRAQ

The remaining 50 µg of the above TRIzol™ protein extracts were resuspended in 8 M Urea in 100 mM Ammonium bicarbonate and were reduced (10 mM DTT) and modified with 40 mM iodoacetamide. The sample was diluted to 2 M urea in water and the proteins were trypsinized with 2 µg bovine trypsin at 37°C overnight. The resulting peptides were cleaned on disposable Silica C18 tip (Harvard) and resuspended in 100 mM Hepes (pH 7.3). The iTRAQ™ Reagent (Applied biosystems) was brought to room temperature and mixed with ethanol (30∶70). After vortexing and spinning, each one of the reagents was transferred to one sample tube. The tubes were incubated at room temperature for 1 h. All four iTRAQ™ reagent labeled samples were combined, cleaned on C18 and resuspended in 0.1% formic acid. Sixty micrograms of the combined labeled peptides were separated in an on-line two dimensional chromatography experiment (MuDPiT). First the peptides were loaded on 15 mm of BioX-SCX column (LC Packing; Dionex, CA, USA)) and eluted with 8 salt steps of 0, 40, 100, 150, 200, 300, 500 and 1000 mM ammonium acetate in 5% ACN and 0.1% acetic acid, pH 3. The eluted peptides were further resolved by capillary reverse-phase chromatography (75µ ID, 30 cm fused silica capillaries, J&W self-packed with 3 µ Reprosil-Aqua C_18_). The peptides were eluted using a 125 min gradient (5% to 40% acetonitrile containing 0.1% formic acid) followed by a wash step of 95% acetonitrile for 15 min. The flow rate was about 0.2 µL /min and the peptides were analyzed by LC-MS/MS on QTOF-Premier mass spectrometer (Waters, Milford, MA, USA). Mass spectrometry was performed in a positive mode using repetitively full MS scan followed by collision induces dissociation (CID) of the 3 most dominant ion selected from the first MS scan. The mass spectrometry data was clustered analyzed and compared using the Pep-Miner searching the human, mouse, rat and bovine part of the NR-NCBI. The identification was done by Sequest search engine, and the quantitation was done it the Libra module from the Trans Proteomics Pipeline [17], comparing the intensity of 114, 115, 116 and 117 ions in each MS/MS spectrum. Each sample was compared to the standard sample B-190 of the same run.

## Results and Discussion

### Primary muscle cultures 2-D PAGE

**Figure 1:**
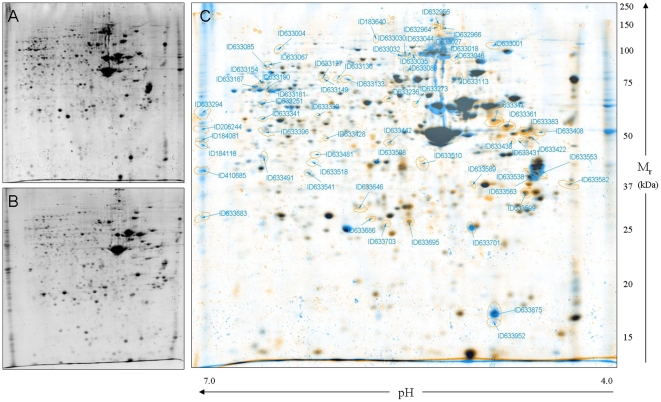
2-DE representative proteomic maps (pH 4.0–7.0) of muscle cultures. A. MS-35 control deltoid muscle; B. MS-312 HIBM deltoid muscle; C. Representative virtual overlay map of controls and HIBM gels; protein expression of control (blue), HIBM (orange) and overlapped expression (black). Only unique (blue or orange) spots were analyzed further.

HIBM is a late onset disease that appears after the muscle tissue in the organism is fully differentiated. Therefore, we conducted proteomic analysis in differentiated cultured myotubes. To our experience each culture has its own differentiation rate, which is not correlated with the disease status of the donor. However, analysis by using anti-MyHC (myosin heavy chain) antibody indicated that the muscle cultures we established in our laboratory are fully differentiated after 5 days in differentiation medium [18]. The matched pairs' characteristics of 3 HIBM and 3 controls primary muscle cultures are presented in Table 1. All the cultures were from deltoid origin except one affected that was gastrocnemius; all samples were clinically affected. In HIBM, deltoid muscle also becomes affected as the disease progresses and the disease pathophysiology is similar in all muscles once they become affected. Although age did not match perfectly between affected patient and control for the last pair (30 versus 46), we think that this age range as well as the entire age range analyzed in this study (Table 1 and Table 2- age range 22–49) still represents subjects at the same stage of life and of muscle biology (young adults). Two of the patients analyzed are of Jewish Persian descent and harbor the founder homozygous mutation at the kinase domain, M712T, and one Persian non Jewish patient (MS-312) carries a homozygous epimerase mutation, V367I [19]. Since muscle culture MS-312 has a different mutation than the other 2 HIBM samples, its inclusion in our analyses should emphasize the disease specific proteomic profile and minimize any mutation specific differences. The cultures were differentiated for 5 days and then incubated for 2–3 more days in free-serum medium in order to prevent sialic acid absorption by the cells. 2-D PAGE of total protein extracts for each sample was performed and achieved a consistent general proteomic pattern on 20 cm gels of pH 4.0–7.0 (Fig. 1A–B). Each HIBM muscle culture proteomic map was compared to its matched control to generate a master proteomic map by PDQUEST 7.3.1 software (Fig. 1C).

In order to focus on disease related proteins, only spots that were found to be differentially expressed (student's test; *p*<0.05) by computerized analysis in all the 3 HIBM samples versus all 3 controls were identified by peptide mass fingerprinting. Each differentially expressed spot was extracted and identified from one HIBM and one control gel to verify that the correct protein spots were compared. The analysis showed 65 differentially expressed spots and 41 proteins were identified; 12 proteins were found to be up-regulated in HIBM myotubes and 29 were down-regulated (Table 3). Data analysis with IPA (Ingenuity Systems) and DAVID (NIH) softwares showed that 31.7% of the differentially expressed proteins are related to cytoskeleton and sarcomere organization (e.g., MYL4, MLRS, & TNNT1). Other functional groups were assigned to ubiquitination (9.7%), glycolysis (7.3%) mitochondrial processes (7.3%) and stress response (4.8%) (Fig. S1). The differential expression ratios were higher than 2 fold for most proteins and western analyses were performed by using selected antibodies to verify the 2-D PAGE proteomic data for few proteins (Fig. S2). The level of troponin-1 and vimentin detected by western blot was in line with the 2-DE analysis results; (vimentin expression was higher in patient samples; troponin-T1 expression was higher in controls); however we could detect only very slight changes between the 2 sample groups in the level of expression of lamin A or heat shock protein 27 in this assay. One explanation could be that the antibodies used detect a different isoform of the protein. Alternatively, several isoforms can be detected, while only one specific isoform was identified from the gel spot. As control proteins we assayed the expression of NCAM, which is a highly polysialylated protein expressed in proliferating satellite cells [11] and VCP, a protein found to be mutated in a myopathy with high similarities to HIBM, inclusion body myopathy associated with Paget disease of bone and frontotemporal dementia (IBMPFD; OMIM 167320) [20]. As expected, no expression differences could be detected in these proteins between the two muscle culture types.

**Table 3:** Differentially expressed proteins in HIBM versus control muscle cultures.

Ref. spot	Description and gene name	Swiss-Prot name/ID	HIBM/Control Fold change	Peptides m/o	Sequence coverage (%)	*p*-value	M_r_ (Da)^a)^	p*I* ^a)^	Possible function
ID633952	Myosin light chain 2; myosin regulatory light chain 2	MLRS/Q96A32	0.28	5/64	7	2.5E-05	19014.7	4.91	Cytoskeleton organization
ID633701	Atrial/embryonic alkali myosin light chain	MYL4/P12829	0.44	21/103	49	0.030	21433.41	4.98	Cytoskeleton organization
ID633133	Tubulin alpha 6	TBA1C/Q9BQE3	0.46	6/84	21	0.010	49895.6	4.96	Cytoskeleton organization
ID633428	Actin alpha cardiac muscle precursor	ACTC/P68032	2.96	12/83	26	0.031	42019.2	5.23	Cytoskeleton organization
ID633599	Tropomyosin 3	TPM3/P06753	2.51	24/95	39	0.002	32818.79	4.68	Cytoskeleton organization, cell motility
ID633361	Vimentin	VIME/P08670	2.03	16/76	44	0.016	53686.0	5.06	Cytoskeleton organization, cell motility
ID633085	Radixin	RADI/P35241	0.34	6/64	12	0.048	68564.2	6.03	Cytoskeleton organization, actin binding
ID633703	Actin, gamma 2 propeptide	ACTH/P63267	2.37	8/84	9	0.020	41877.1	5.31	Cytoskeleton organization, actin signaling
ID633154	WD repeat-containing protein 1 isoform 2	WDR1/O75083	0.28	5/61	7	0.018	58002.6	6.41	Cytoskeleton organization, actin assembly
ID633113	Kelch repeat and BTB (POZ) domain	KBTBA/O60662	0.46	14/75	25	0.010	66791.4	5.49	Muscle contraction
ID633541	Troponin T1	TNNT1/P13805	0.25	8/82	9	0.004	32934.3	5.85	Muscle contraction, calcium signaling
ID633032	Nexilin	NEXN/Q0ZGT2	0.13	2/108	3	0.009	80658.28	5.31	F actin binding, stress fibers
ID633067	Zyxin	ZYX/Q15942	0.26	4/72	12	0.004	61277.7	6.22	Cell adhesion, α- actinin binding protein
ID206244	Proteasome 26S ATPase subunit 5	PRS8/P62195	0.10	6/93	19	0.015	45626.3	7.11	Ubiquitination pathway
ID633695	Ubiquitin carboxyl-terminal esterase L1	UCHL1/P09936	2.18	5/69	28	0.008	24824.5	5.33	Ubiquitination pathway
ID633442	COP9 signalosome subunit 4	CSN4/Q9BT78	2.24	7/71	20	0.027	46269.1	5.57	Ubiquitination pathway
ID633046	Proteasome 26S non-ATPase subunit 2	PSMD2/Q13200	0.47	13/87	19	0.039	100200.3	5.08	Ubiquitination pathway, proteosome
ID633001	Enolase 3	ENOB/P13929	0.10	5/93	12	0.006	46959.1	7.58	Glycolysis
ID633004	2-oxoglutarate dehydrogenase	ODO1/Q02218	0.20	12/59	10	0.001	113478.1	6.62	Glycolysis
ID633347	Enolase 2	ENOG/P09104	2.29	4/76	5	0.015	47268.8	4.91	Glycolysis
ID633294	ATP synthase	ATPA/P25705	0.15	10/80	26	0.024	59750.9	9.16	Oxidative phosphorylation
ID633089	NADH dehydrogenase	NDUS1/P28331	0.26	11/89	23	0.004	79468.0	5.89	Oxidative phosphorylation
ID633251	Aldehyde dehydrogenase 1A1	AL1A1/P00352	0.22	12/96	25	0.010	54862.1	6.30	Electron transport, alcohol metabolism
ID633422	Reticulocalbin 1	RCN1/Q15293	2.29	7/61	25	0.041	38890.2	4.86	Calcium binding
ID633408	Reticulocalbin 3	RCN3/Q96D15	3.25	8/92	41	0.010	37493.2	4.74	Calcium binding, ER
ID633167	Stress-induced-phosphoprotein 1	STIP1/P31948	0.46	9/81	13	0.003	62639.6	6.40	Stress response
ID633686	Heat shock 27kD protein 1	HSPB1/P04792	5.42	5/56	25	0.016	22782.6	5.98	Stress response to unfolded proteins
ID633127	Vesicle transport-related protein isoform a	SCFD1/Q8WVM8	0.29	6/72	16	0.003	72380.2	5.89	Vesicle mediated transport, ER to Golgi
ID633030	Coatomer protein complex, subunit gamma 1	COPG/Q9Y678	0.17	11/73	14	6.9E-05	97718.8	5.32	Vesicle mediated transport, Golgi to ER
ID633001	RAN binding protein 5	IPO5/O00410	0.38	6/82	5	0.006	125545.8	4.80	Protein import to nucleus
ID633133	Nuclear VCP-like	NVL/O15381	0.36	6/65	9	0.010	82746.6	6.04	ATP binding triphosphatase activity
ID633294	Glutamate dehydrogenase 1	DHE3/P00367	0.15	7/80	16	0.024	61398.2	7.66	AA metabolism
ID633154	EH-domain containing 4	EHD4/Q9H223	0.28	9/61	15	0.018	61175.5	6.33	AA metabolism
ID633646	6-phospho- gluconolactonase	6PGL/O95336	2.13	7/88	41	0.001	27547.0	5.70	Carbohydrate metabolism
ID633646	Peroxisomal long-chain acyl-coA thioesterase	ACOT2/P49753	0.10	6/93	15	0.001	53265.9	8.82	Fatty acid metabolism
ID633236	Dihydropyrimidinase-like 2	DPYL2/Q16555	3.08	5/97	11	0.019	62294.0	5.95	Cell communication
ID410685	Cyclin-dependent kinase 3	CDK3/Q00526	0.14	4/71	16	0.008	35045.9	8.86	Cell cycle
ID633149	Protein arginine methyltransferase 5 isoform a	ANM5/O14744	0.24	11/114	18	0.002	72684.3	5.88	Cell proliferation
ID633341	Lamin A/C	LMNA/P02545	0.25	11/54	21	0.017	65135.1	6.40	Apoptosis signaling
ID633127	PMF-1 binding protein	PMFBP/Q8TBY8	0.29	10/109	13	0.003	117480.4	5.90	Cell death
ID633341	Protein phosphatase 2 isoform b	2AAB/P30154	0.46	7/75	11	0.017	73585.2	4.96	Cell death

Theoretical p*I* and M_r_ values were calculated using the ExPASY tool. m = matched peptides, o = observed peptides.

To note, in spite of the differences seen in the expression of several sarcomeric proteins between HIBM and control cell cultures that could anticipate that HIBM derived cells would present some defect of development, no differences could be detected in these cultures in our previous studies [18] in terms of morphology, proliferation rate or differentiation processes, except for their response to apoptosis through the AKT pathway.

### 2-D PAGE analysis of muscle specimens

For 2D PAGE analysis, skeletal muscle specimens were obtained from HIBM patients who harbored the founder homozygous mutation M712T (Table 2). Five of the 6 samples (3 pairs of HIBM/controls) were deltoid muscle, one affected was tibialis anterior. As seen in Fig. S3, all 3 affected muscles (2 deltoids and one tibialis anterior) presented typical HIBM pathology, at a similar stage.

**Figure 2:**
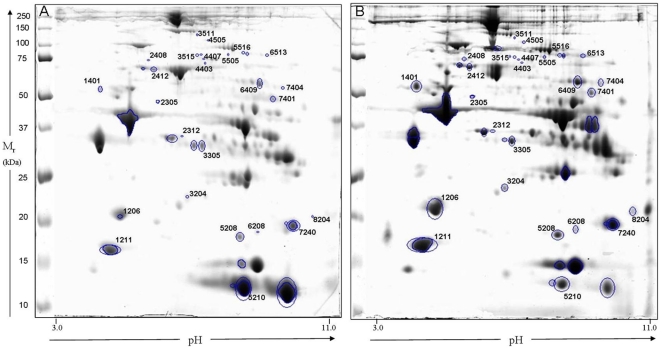
2-DE proteomic maps (pH 3.0–11.0) of A. B-270 control deltoid muscle and B. B-147 HIBM deltoid muscle.

2-DE proteomic maps of three HIBM biopsies and their matched controls were generated using 20 cm gels over a pH range of 3.0–11.0. Each HIBM biopsy was compared to its matched control by PDQUEST 7.3.1 software (Fig. 2) and only spots significantly differentially expressed (student's test; *p*<0.05) in all 3 HIBM samples versus all 3 controls were identified by MS/MS spectra. Peptides from 34 extracted spots were related to 26 different proteins by using Sequest and Pep-miner softwares with comparison to the human part of NR-NCBI database (Table 4, Table S1). When similar proteins were identified at different spots their altered expression was always in the same direction and therefore, it is not likely that a certain isoform or post-translational modification is more abundant than the other in one of the sample groups. Computerized analysis revealed that the expression of 78% of altered proteins was upregulated in HIBM biopsies and that, in accordance with the muscle culture results, the main cluster of differentially expressed proteins was attributed to cytoskeleton and sarcomere organization functions (46.1%) (e.g., MYL3, MYH7, TNNI1 & TNNT1). Other functional groups of proteins were related mainly to oxidative phosphorylation (NDUS1, ATPA & QCR1), stress response (HSPB1, HSP7C & CRYAB) and glycolysis (ODO1, KPYM & K6PF), each of them representing 13-11.5% of all the identified differential proteins (Fig. S4).

**Table 4:** Differentially expressed proteins in HIBM versus control muscle biopsies as determined by 2-DE analysis.

Ref. spot	Description and gene name	Swiss-Prot name/ID	HIBM/Control Fold change	Sequence coverage (%)	Theoretical M_r_ (Da)a)	Theoretical p*I* a)	No. of confirmatory MS/MS peptides	Possible function
1206	Slow skeletal ventricular myosin alkali light chain 3	MYL3/P08590	4.95	78	21800.87	5.03	19	Cytoskeleton organization
3515	Myosin heavy chain 7 cardiac muscle, beta	MYH7/P12883	2.90	25	223733.72	5.60	41	Cytoskeleton organization
1401	Actin alpha cardiac muscle precursor	ACTC/P68032	2.01	53	41784.64	5.23	12	Cytoskeleton organization
1211	Myosin light chain 2	MLRS/Q96A32	1.28	86	18883.38	4.91	13	Cytoskeleton organization
3511	Vinculin isoform VCL	VINC/P18206	1.92	65	123668.11	5.51	63	Cytoskeleton organization
1401	Tropomyosin 1 alpha chain isoform 1	TPM1/P09493	7.43	87	32708.57	4.69	48	Cytoskeleton organization
4407	Moesin	MOES/P26038	15.27	39	67688.85	6.09	25	Cytoskeleton organization
7404	Myotilin	MYOTI/Q9UBF9	3.92	44	55395.14	9.12	20	Cytoskeleton organization
4403	Radixin	RADI/P35241	1.10	42	68563.90	6.03	23	Cytoskeleton organization
8204	Troponin I, skeletal, slow	TNNI1/P19237	46.12	49	21561.15	9.61	13	Muscle contraction
3305	Troponin T, slow skeletal muscle	TNNT1/P13805	1.45	36	32816.97	5.86	15	Muscle contraction
7240	Troponin I, skeletal, fast	TNNI2/P48788	0.69	58	21207.33	8.88	16	Muscle contraction
2412	Heat shock 70kDa protein 8 isoform 1	HSP7C/P11142	1.60	61	70766.90	5.37	41	Stress response to unfolded proteins
3204	Heat shock 27kDa protein1	HSPB1/P04792	1.49	47	22782.52	5.98	11	Stress response to unfolded proteins
5208	Crystallin, alpha B	CRYAB/P02511	1.22	71	20158.91	6.76	15	Stress response to unfolded proteins
7401	ATP synthase, H+ transporting, mitochondrial	ATPA/P25705	1.83	51	55209.32	8.28	32	Oxidative phosphorylation
2408	NADH dehydrogenase (ubiquinone) Fe-S protein1	NDUS1/P28331	2.26	53	79467.50	5.89	30	Oxidative phosphorylation
2305	Ubiquinol-cytochrome c Reductase core protein I	QCR1/P31930	2.20	48	52645.82	5.94	21	Oxidative phosphorylation
2312	Cytosolic malate dehydrogenase	MDHC/P40925	0.51	33	36294.93	6.89	10	Carbohydrate metabolism
5516	Aconitase 2	ACON/Q99798	2.35	40	85425.41	7.36	27	Carbohydrate metabolism
4505	Oxoglutarate dehydrogenase isoform	ODO1/Q02218	1.74	44	115935.28	6.39	42	Glycolysis
6409	Pyruvate kinase	KPYM/P14618	1.25	73	57805.70	7.95	44	Glycolysis
6513	Phosphofructokinase, muscle	K6PF/P08237	3.68	21	85051.33	8.23	14	Glycolysis
5505	Adenosine monophosphate deaminase 1	AMPD1/P23109	2.20	58	86489.87	6.43	38	Purine metabolism
6208	Prostatic binding protein	PEBP1/P30086	4.40	57	20925.59	7.43	9	Signal transduction in nervous system
5210	Beta globin	HBB/P68871	0.60	96	15867.22	6.81	14	Hemoglobin polymerization

a) Theoretical p*I* and M_r_ values were calculated using the ExPASY tool.

### iTRAQ analysis of muscle specimens

**Figure 3:**
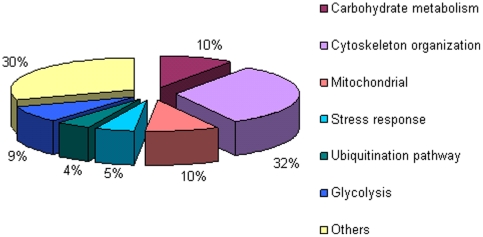
Functional annotations of the differentially expressed proteins in HIBM versus controls as analyzed by 2-DE of muscle cultures and 2-DE of biopsies and iTRAQ of biopsies.

The same biopsies protein extracts that were subjected to 2-D PAGE above were also relatively quantified by iTRAQ (Table 5). In addition, in an attempt to determine as precisely as possible the specificity of the proteome of HIBM versus other clinically, pathogenically and/or histologically related muscle disorders, we included in this analysis 4 biopsy samples of Welander distal myopathy (WDM; OMIM 604454), Tibial muscular dystrophy (TMD; OMIM 600334) and Distal myopathy type 3 (MPD3; OMIM 610099) that exhibit closely related features to HIBM [21]–[23] (Table 5). In each run four samples were tested including the control sample B-190 that was used as internal standard. Each sample's tryptic peptides were uniquely mass tagged and mixed with their matched pair sample. The peptides mixture was then separated in 2D LC-MS/MS and the data was analyzed with the Pep-Miner software searching against the NR-NCBI database. About 400 proteins were identified, in each sample, however no protein was found to be uniquely present in all HIBM or control samples. The Wilcoxon signed-rank test revealed 41 proteins (Table 6, Table S2) that were most differentially expressed in all 3 HIBM samples versus all 3 controls, however this difference was not statistically significant (*p* = 0.1090). Comparison of these 41 proteins between the 3 control samples versus all 4 samples of non HIBM myopathies by Mann-Whitney test showed significant expression differences (*p* = 0.037) only for four of them: ALBU, TPM2, CASQ1 and ANKR2. These proteins could represent more common or downstream pathways for distal myopathies and are probably not specific solely to the HIBM pathophysiology. Thus, the 37 remaining differential expressed proteins may be considered as HIBM specific, at least among these distal myopathies. Functional annotation analyses of these proteins by DAVID and IPA softwares coincide with the 2-D PAGE findings of both muscle biopsies and myotubes cultures and show that gene products differentially expressed in HIBM versus controls are involved in carbohydrate metabolism (17.0%), mitochondrial processes (12.1%) and glycolysis (12.1%) but the main cluster represents again proteins involved in cytoskeleton and sarcomere organization (29.2%) (Fig. S5). Taken together from the 2-DE and the iTRAQ analyses, 61 proteins were differentially expressed and the majority of them showing less than 2 fold changes. Although only few proteins overlapped in all 3 assays, overall the largest group of altered proteins in all 3 comparisons analyses was related to cytoskeleton and sarcomere organization (Fig. 3).

**Table 5:** Biopsies samples used in the different iTRAQ runs.

Run	HIBM	Controls	Other distal myopathies	Internal standard
**1**	B-147 Del, M (45)	B-270 Del, M (48)	TMD TA, M (61)	B-190 Del, M (32)
**2**	B-220 TA, F (22)	B-181 Del, M (22)	MPD3 TA, M (37)	B-190 Del, M (32)
**3**	B-171 Del, F (35)	B-190a) Del, M (32)	MPD3 TA, M (44)WDM TA, F (60)	B-190a) Del, M (32)

a) In run 3 sample B-190 was used both as control and as internal standard.

Del = Deltoid, TA = Tibialis Anterior, M = Male, F = Female and age of patient (years).

**Table 6:** Differentially expressed proteins in HIBM versus control muscle biopsies as determined by iTRAQ analysis.

Description and gene name	Swiss-Prot name/ID	HIBM/Control Fold change	Sequence coverage (%)	Theoretical M_r_ (Da)a)	Theoretical p*I* a)	No. of confirmatory MS/MS peptides	Possible function
Myosin light polypeptide 6B	MYL6B/P14649	0.53	51	22763.99	5.56	11	Cytoskeleton organization
Myosin heavy chain 3	MYH3/P11055	0.70	19	223904.73	5.62	3	Cytoskeleton organization
Myosin heavy chain 2	MYH2/Q9UKX2	3.08	57	223044.46	5.64	68	Cytoskeleton organization
Myosin light chain 2	MLRS/Q96A32	2.2	88	18883.38	4.91	23	Cytoskeleton organization
Myosin heavy chain 4	MYH4/Q9Y623	1.63	33	223071.41	5.65	6	Cytoskeleton organization
Alpha-actinin-2	ACTN2/P35609	1.28	61	103853.79	5.31	62	Cytoskeleton organization
Tropomyosin alpha-1 chain	TPM1/P09493	2.96	54	32708.57	4.62	17	Cytoskeleton organization
Tropomyosin beta chain	TPM2/P07951	1.72	60	32850.73	4.66	19	Cytoskeleton organization
Myomesin-2	MYOM2/P54296	1.32	15	164896.31	5.82	17	Cytoskeleton organization
Tubulin beta chain	TBB5/P07437	1.49	11	49670.82	4.78	4	Cytoskeleton organization
Troponin C, skeletal muscle fast	TNNC2/P02585	2.06	45	17990.86	4.06	7	Muscle contraction
Troponin I, fast skeletal muscle	TNNI2/P48788	2.33	33	21207.33	8.88	12	Muscle contraction
Ankyrin repeat domain-containing protein 2	ANKR2/Q9GZV1	1.12	23	39859.25	5.72	6	Muscle stress response
Sarcalumenin	SRCA/Q86TD4	1.20	8	48692.49	3.81	5	Calcium transport
Protein S100-A4	S10A4/P26447	0.74	27	11597.32	5.88	4	Tubulin Polymerization
Pyruvate kinase isozymes M1/M2	KPYM/P14618	1.58	55	57805.70	7.95	26	Glycolysis
Gamma-enolase	ENOG/P09104	1.62	19	47137.39	4.91	1	Glycolysis
Glucose-6-phosphate isomerase	G6PI/P06744	1.50	9	63015.93	8.44	5	Glycolysis
Alpha-enolase	ENOA/P06733	1.12	24	47037.77	6.99	4	Glycolysis
Triosephosphate isomerase	TPIS/P60174	1.38	85	26538.30	6.51	19	Glycolysis
Malate dehydrogenase, mitochondrial precursor	MDHM/P40926	1.06	29	35503.28	8.92	9	Carbohydrate metabolism
Malate dehydrogenase, cytoplasmic	MDHC/P40925	0.91	41	36294.93	6.89	12	Carbohydrate metabolism
Phosphoglucomutase-1	PGM1/P36871	1.68	23	61317.93	6.32	13	Carbohydrate metabolism
Glycogen debranching enzyme	GDE/P35573	1.64	11	174763.74	6.31	13	Carbohydrate metabolism
Aldose reductase	ALDR/P15121	1.18	16	35722.21	6.55	5	Carbohydrate metabolism
Glycogen phosphorylase	P11217/PYGM	1.78	59	96960.86	6.57	48	Carbohydrate metabolism
Glyceraldehyde-3-phosphate dehydrogenase	G3P/P04406	1.42	66	35922.02	8.58	34	Carbohydrate metabolism
Cytochrome c oxidase subunit 4	COX41/P13073	1.11	24	19576.71	9.52	4	Oxidative phosphorylation
Calsequestrin-1	CASQ1/P31415	3.31	21	41688.88	3.96	6	Mitochondrial calcium binding
Dihydrolipoyl dehydrogenase	DLDH/P09622	0.85	9	54177.25	7.95	3	Mitochondrial glycine cleavage
Hydroxyacyl-coenzyme A dehydrogenase,	HCDH/Q16836	0.87	11	34277.50	8.88	3	Mitochondrial oxidation
Cytochrome b-c1 complex	UCRI/P47985	1.37	19	29667.99	8.55	3	Mitochondrial oxidation
Peroxiredoxin-6	PRDX6/P30041	1.45	34	24903.79	6.02	7	Redox regulation
Histidine triad nucleotide-binding protein 1	HINT1/P49773	1.13	51	13670.72	6.46	4	Nucleotide binding
Probable C→U-editing enzyme APOBEC-2	ABEC2/Q9Y235	1.53	46	25703.14	4.81	7	mRNA editing
Phosphatidylethanolamine-binding protein 1	PEBP1/P30086	1.27	42	20925.59	7.43	5	Signal transduction in nervous system
Acyl-CoA-binding protein	ACBP/P07108	1.11	20	9913.23	6.11	2	Lipid metabolism
Serum albumin precursor	ALBU/P02768	1.49	68	66472.21	5.67	54	Fatty acid & steroid transport
Glycerol-3-phosphate dehydrogenase	GPDA/P21695	1.69	20	37436.44	5.82	6	Glycerol metabolism
Alpha-1-antitrypsin precursor	A1AT/P01009	0.78	20	46736.55	5.37	7	Protease inhibition
Aspartate aminotransferase, mitochondrial	AATM/P00505	1.43	25	47475.57	9.14	9	AA metabolism

a) Theoretical p*I* and M_r_ values were calculated using the ExPASY tool.

### HIBM proteome in biopsies versus muscle cultures

Most differentially expressed proteins (85%) were overexpressed in HIBM biopsies, although the expression ratios were lower than 2 fold. The low differential ratios obtained from the biopsies analysis are consistent with our previous GeneChip HIBM biopsies expression microarrays studies that also demonstrated less than 2 fold change in most of the differential transcripts detected [24] and possibly maybe expected in a late onset slowly progressive disease. In contrast, and in spite of the fact that no changes were observed in either proliferation, differentiation or senescence patterns [18] and no organization aberrations were detected when the cells were grown in 3D cultures (Mitrani-Rosenbaum et al., unpublished observations), about 70% of the proteins found to be differentially expressed in the disease cultured myotubes were downregulated. In addition, most of the differential proteins ratios in the myotube cultures were higher than 2 fold. To explain this issue, one should consider the nature of the samples analyzed: biopsies represent mature muscle tissue, organized as sarcomeres, and probably reflect the behavior of the organism in terms of global physiology, so that the overexpression of many proteins seen in the biopsies analysis could result from the compensatory mechanisms taking place in the muscle tissue as a whole, in an attempt to maintain the tissue organization that confers to muscle its function. In contrast, muscle cultures components are solely “isolated” satellite cells, and therefore could reflect only the basic and maybe more primary cellular defects occurring in HIBM, such as down regulation of many proteins. Even though, the differential proteins found in the analysis of both muscle cultures and biopsies were related to the same several functional groups, assigned to mitochondrial processes, stress response and ubiquitination, the main cluster being represented by proteins involved in cytoskeleton and sarcomere organization. Therefore, as a whole, it seems reasonable to use HIBM primary muscle cultures as a model to investigate the fine molecular cascades that lead to the pathology of the disease. In these studies we have also compared 2 techniques for proteomic analysis, by using the same biopsies protein extracts in 2-D PAGE and iTRAQ analyses. By both analyses, the biological pathways unraveled in HIBM pathophysiology were the same, although the expression fold figures were much higher in the 2-DE analysis than in the iTRAQ one, even for the very same protein samples (Tables 4 and 6). One possible explanation could be that in a specific 2-DE gel spot which is quantified, very often more than one protein could be detected, while in the iTRAQ technique, proteins and peptides are separated much more efficiently.

### Changes in the expression of cytoskeleton and sarcomeric proteins

The correct organization and structure of the muscle cytoskeleton and sarcomeric proteins are keys to its function and to the maintenance of its integrity during contraction. This group of proteins is highly abundant in muscles and indeed found to be the major cluster of proteins with altered expression in HIBM by all 3 analyses described here (30%). Similar proteomics studies have been performed only for few adult onset myopathies. Sporadic Inclusion Body Myositis (s-IBM), belonging to the inflammatory group of such diseases and presenting many pathological similarities with HIBM, was shown to display slightly disturbed expression patterns of sarcomeric proteins [25] with a switch from fast to slow twitch type fibers [26] that was previously hypothesized based on immunohistochemistry data [27]. Proteomic clues of such a switch were also found in a limb girdle myopathic disorder, dysferlinopathy [28]. In contrast, our data showed disturbed cytoskeleton and sarcomeric proteins expression patterns but no trend was found with regard to the different slow/fast isoforms expression. Although there is no data describing a different distribution of slow and fast twitch type fibers in those muscles, it has to be noted that we have focused on deltoid muscle, whereas the above mentioned studies examined quadriceps and biceps. Nevertheless, fast to slow twitch fiber type switch occurs also in healthy muscle due to disuse [29] or sarcopenia [30]–[31] and therefore these parameters should be taken into consideration when determining whether it is a direct consequence of muscle disease. Thus, among the myopathies analyzed by proteomics to date, it could be that changes in the major cluster of cytoskeletal and sarcomeric proteins are specific to HIBM. These findings raise the issue of the role of GNE in filament organization and maintenance. Recently we have shown that GNE interacts with α-actinin 1, an actin binding protein, at the M line and juxtaposed to the Z line in mouse muscle [32]. Humans harbor 4 α-actinin isoforms ,α-actinin 2 and α-actinin 3 are known as muscle proteins located in the Z-disk and crosslink actin filaments to stabilize the sarcomere during muscle contraction [33]. Notably, additional proteins found in this study as differentially expressed in HIBM are also involved in the sarcomeric organization of muscle fibers: zyxin, an actinin binding protein was downregulated in HIBM muscle whereas α-actinin1 precursor and α-actinin 2 are upregulated, as well as proteins related to calcium binding such as reticulocalbin 3, reticulocalbin 1 precursor, sarcalumenin and calsequestrin. Interestingly, calcium related proteins were found to be downregulated in Duchenne muscular dystrophy where dystrophin mutations are believed to destabilize the major sarcolemma dystrophin-glycoprotein complex which in turn affects sarcolemma integrity and calcium buffering [34], [35]. Our results are in line with the premise that HIBM is not a disorder of the sarcolemma In addition the hypothesis that GNE plays a role in the organization of the sarcomere finds further support in the many features shared by HIBM and tibialis muscular dystrophy (TMD), a late onset progressive autosomal disease characterized by weakness in the anterior lower leg (in particular the tibialis anterior) and rimmed vacuoles. TMD is caused by mutations in titin, a large protein that serves as an adhesion template for the assembly of the sarcomere in muscle [22]. Further studies are required in order to examine the interaction between GNE and the α-actinin isoforms and to determine whether GNE has a novel function directly involved in sarcomere filament organization.

### Changes in the expression of mitochondrial and glycolysis proteins

Additional outstanding differentially expressed functional clusters are related to mitochondrial processes such as oxidative phosphorylation, electron transport and to glycolysis. Clues for mitochondria functions impairment in HIBM were also found previously in genomic expression patterns comparison of muscle specimens from 10 HIBM patients carrying the M712T mutation and presenting mild histological changes, compared with 10 healthy matched control individuals [24]. A strikingly high proportion (18.6%) of the overall differentially expressed mRNAs of known function were found to encode for proteins implicated in various mitochondrial processes, revealing mitochondria pathways dysregulation specific to HIBM. Mitochondria are the essential organelles for energy supply of cells and can respond to multiple physiological stresses via signaling processes, cell growth and differentiation events. In particular, they play a major role in cell death as sensors of apoptotic signals, by releasing various pro-apoptotic molecules into the cell cytoplasm. The precise events upstream to mitochondria apoptotic involvement remain to be fully characterized, however it is well accepted that Akt is a key factor in this apoptotic/cell survival process. Notably, our previous findings of a primary impairment of apoptotic events and survival defects in HIBM cells, as shown by the lack of activated Akt response to apoptotic stimuli [18], are in line with the mild mitochondrial alterations described here. To note, in sarcopenia, mitochondrial activity is enhanced and glycolysis reduced, probably as a reflection of a more intensive aerobic oxidative metabolism with age [36], whereas our results point to a more intense activity of both mitochondrial and glycolysis proteins, thus probably indicating compensatory effects of the degenerating mature fiber in HIBM.

### Changes in the expression of stress proteins

Inclusion bodies in HIBM have been related to an unfolded protein response through endoplasmic reticulum and oxidative stress proteins response mechanisms [37]. Over expression of ubiquitin and proteosome proteins in HIBM [26] and the recent identification of similar protein aggregates in an HIBM mouse model, along with the overexpression of GRP94, support this hypothesis [38]. In our present analyses of both cultures and biopsies we found 10 differentially expressed proteins related to stress response and to the ubiquitin pathway, among them ANKR2, HSP7C, HSPB1 & CRYAB that are upregulated in HIBM samples. Increased chaperone expression was also found in other muscular dystrophies and senescence [39] and could, therefore, represent downstream processes common to those pathologies, such as enhanced functioning of repair mechanisms to remodel the degenerating fibers. None of the reported aggregated proteins, tau and beta amyloid in particular, were detected in our studies; however, this might be due to poor solubilization.

Proteomic studies on muscle cultures and biopsies from HIBM specimens, by 2-DE and iTRAQ analyses, emphasize changes in proteins of the cytoskeleton and the sarcomere filaments, affecting its maintenance and organization. These findings could point to a role of GNE in the muscle filamentous apparatus that could contribute to HIBM pathogenesis.

The altered expression levels of distinct skeletal muscle proteins, as documented in this study, might be helpful not only for deepening our understanding of the pathophysiology of HIBM but also for the future establishment of a comprehensive biomarker signature of the disease. Reliable markers could be used for improving diagnostics, monitoring of disease progression and therapeutic evaluations.

## Supporting Information

Figure S1 **Functional annotations of the differentially expressed proteins in HIBM versus controls as analyzed by 2-DE of muscle cultures.**
(TIF)

Figure S2 **Western analysis of two matched HIBM/ control muscle culture pairs with antibodies for representative proteins.**
(TIF)

Figure S3 **Histological sections of biopsies analyzed by 2D gels and iTraq.** a–c: HIBM muscle samples; d–f, normal controls. Arrows point to degenerating fibers with rimmed vacuoles, showing a similar stage of typical HIBM pathology in all 3 affected samples; a) tibialis anterior; b–f, deltoid.
(TIF)

Figure S4 **Functional annotations of the differentially expressed proteins in HIBM versus controls as analyzed by 2-DE of biopsies.**
(TIF)

Figure S5 **Functional annotations of the differentially expressed proteins in HIBM versus controls as analyzed by iTRAQ.**
(TIF)

Table S1 **MS/MS identification of protein biopsies by 2DE.**
(XLS)

Table S2 **MS/MS identification of protein biopsies by iTRAQ.**
(XLS)
