# Supplementary figures and images for: The Proteomic Profile of Hereditary Inclusion Body Myopathy
Source: PLoS One. 2011 Jan 31;6(1):e16334. doi: 10.1371/journal.pone.0016334 (PMC3031555; doi:10.1371/journal.pone.0016334)

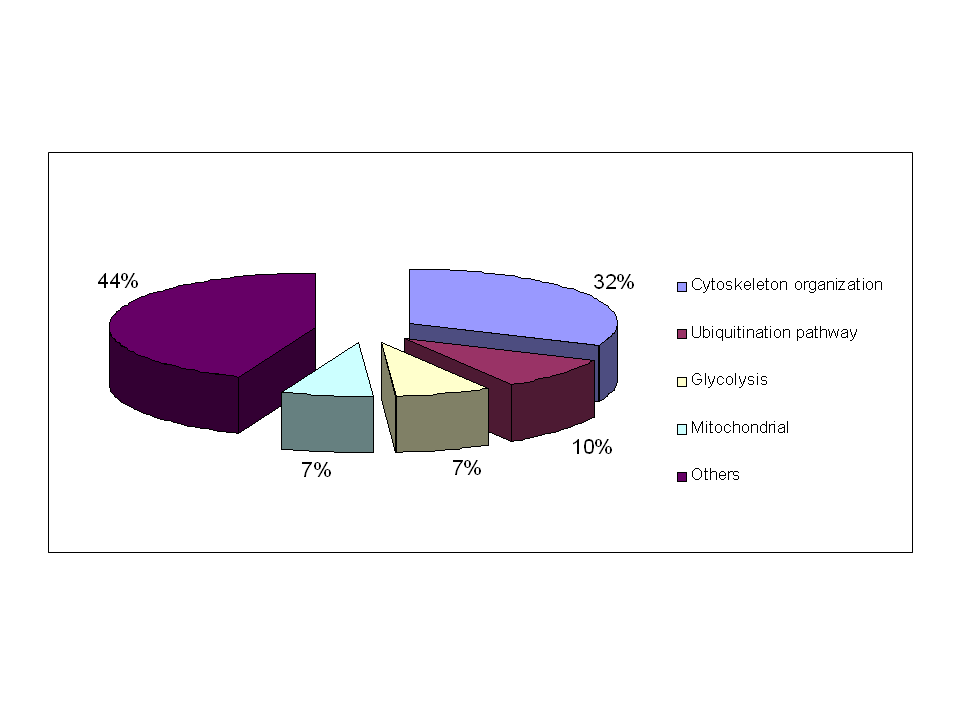

Supplement: Figure S1 — Functional annotations of the differentially expressed proteins in HIBM versus controls as analyzed by 2-DE of muscle cultures. (TIF) [file pone.0016334.s001.tif]

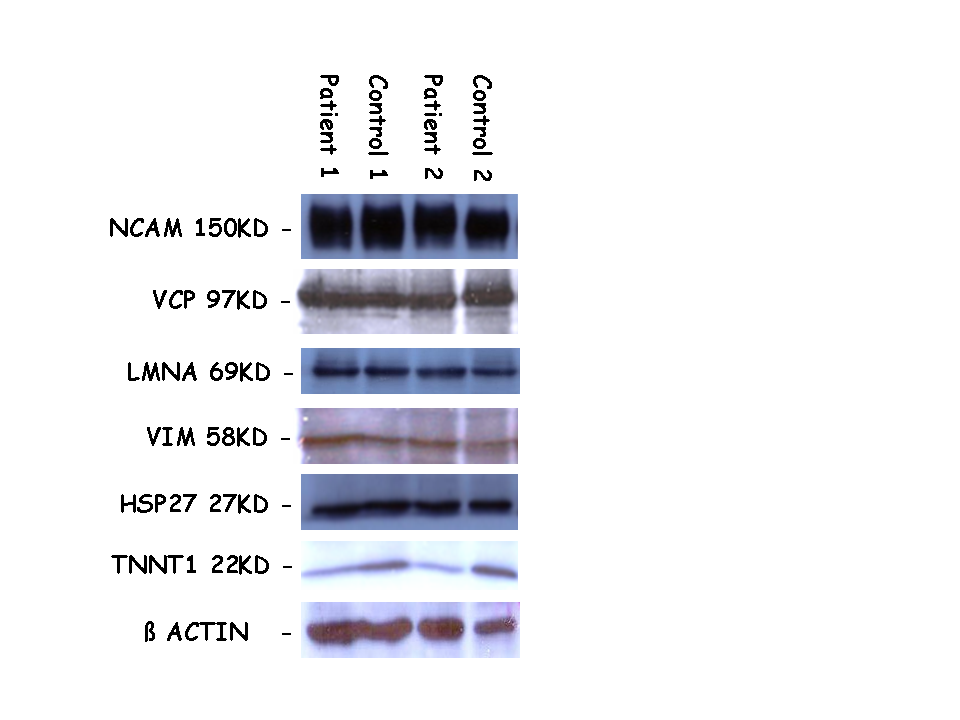

Supplement: Figure S2 — Western analysis of two matched HIBM/ control muscle culture pairs with antibodies for representative proteins. (TIF) [file pone.0016334.s002.tif]

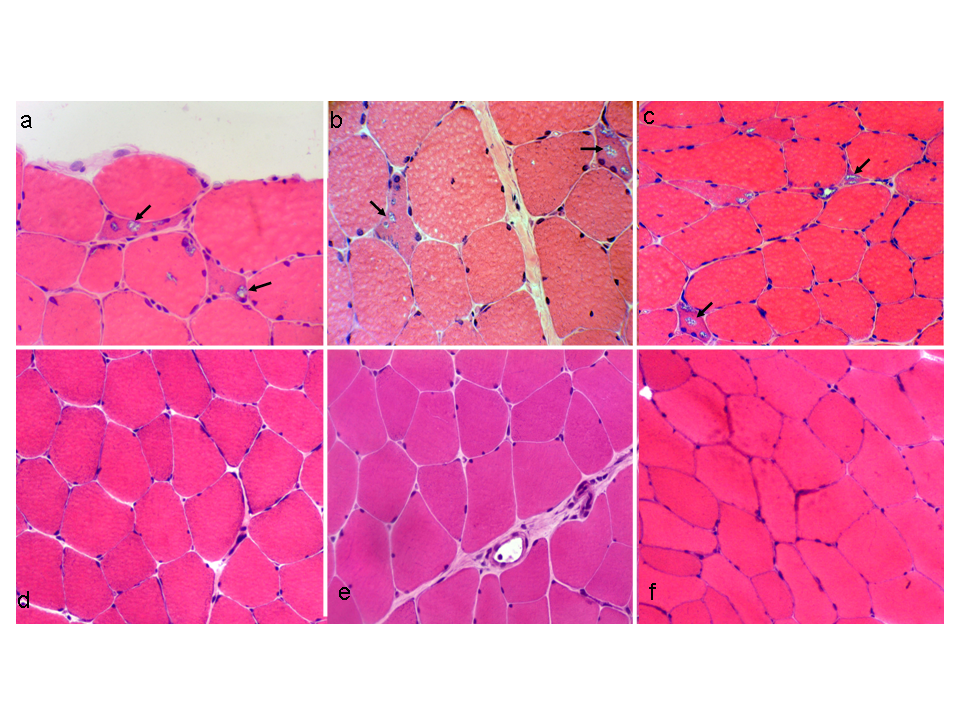

Supplement: Figure S3 — Histological sections of biopsies analyzed by 2D gels and iTraq. a–c: HIBM muscle samples; d–f, normal controls. Arrows point to degenerating fibers with rimmed vacuoles, showing a similar stage of typical HIBM pathology in all 3 affected samples; a) tibialis anterior; b–f, deltoid. (TIF) [file pone.0016334.s003.tif]

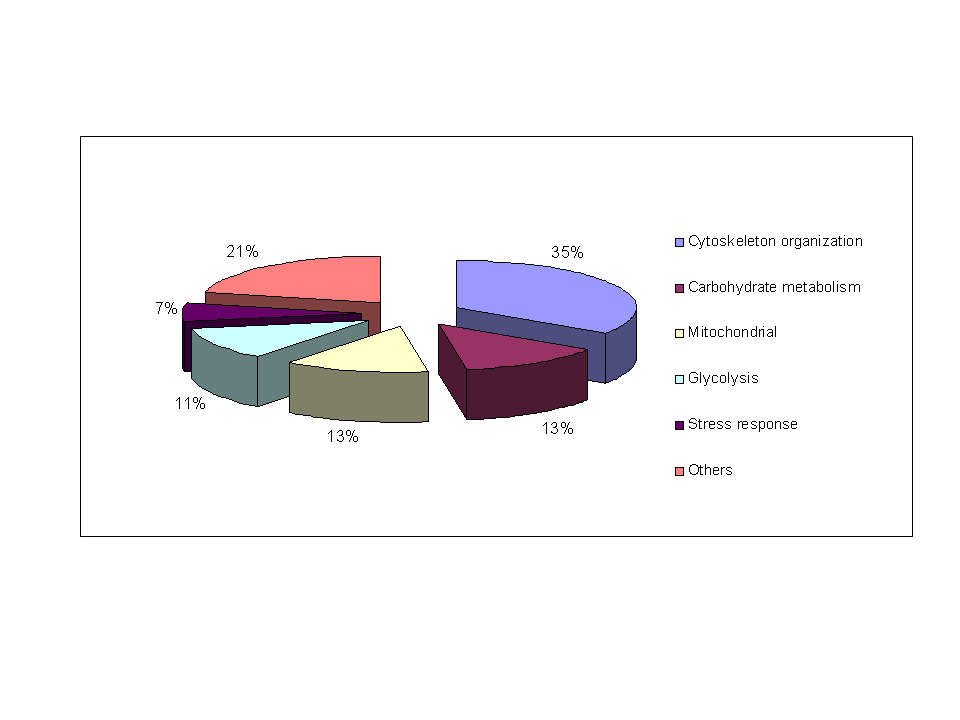

Supplement: Figure S4 — Functional annotations of the differentially expressed proteins in HIBM versus controls as analyzed by 2-DE of biopsies. (TIF) [file pone.0016334.s004.tif]

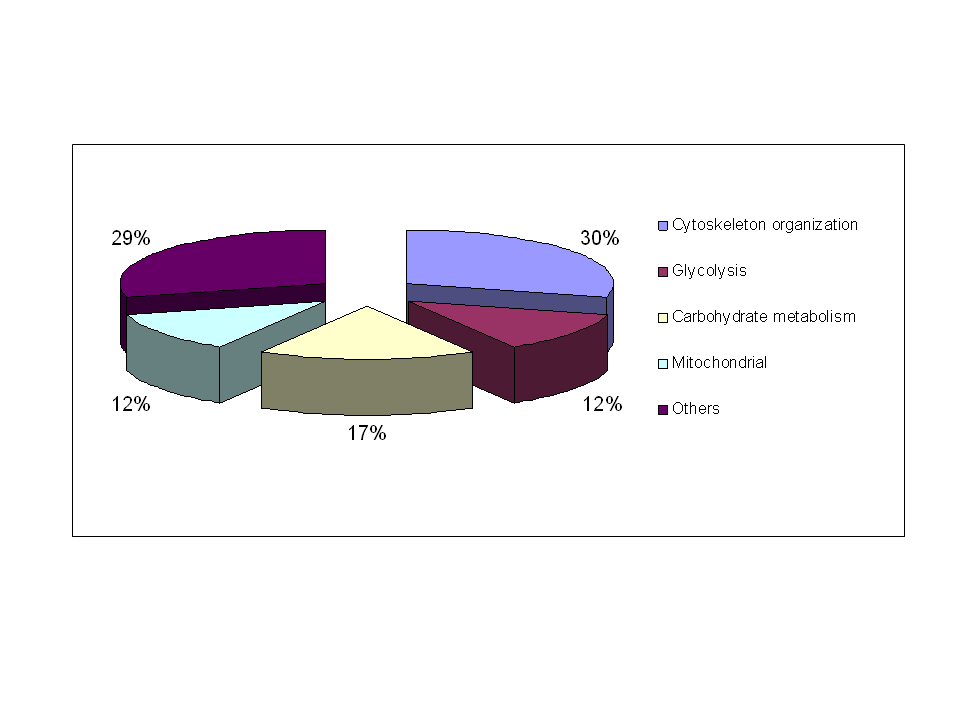

Supplement: Figure S5 — Functional annotations of the differentially expressed proteins in HIBM versus controls as analyzed by iTRAQ. (TIF) [file pone.0016334.s005.tif]
